# Body weight misperception and psychological distress among young South Korean adults: the role of physical activity

**DOI:** 10.1186/s41256-017-0036-9

**Published:** 2017-06-05

**Authors:** Eun-Young Lee, Maxine Myre, Jongnam Hwang, Heeran Chun, Eunchul Seo, Roman Pabayo, John C. Spence

**Affiliations:** 1 Faculty of Physical Education and Recreation, University of Alberta, 1-149 Van Vliet Complex, Edmonton, AB Canada; 2 Department of Health Promotion, Daegu University, Daegu, South Korea; 3 College of Health Sciences, Jungwon University, Geosan, Chung-buk South Korea; 4 Department of Sport Science, Dankook University, Cheonan, South Korea; 5 School of Community and Health Sciences, University of Nevada, Reno, NV USA; 6 Harvard School of Public Health, Department of Social and Behavioral Sciences, Boston, MA USA

**Keywords:** Body weight, Stress, Suicidal ideation, Depression, Physical inactivity, KNHANES

## Abstract

**Background:**

Emerging evidence suggests that body weight misperception may be associated with psychological distress among people in developed countries. Participating in physical activity (PA) may negate the association between weight misperception and psychological distress given the well-known benefits of PA on psychological health. This study examined the role of PA in associations between body weight misperception and psychological distress among young South Korean adults.

**Methods:**

Data from individuals aged 20 to 39 years who participated in the Fifth Korean National Health and Nutrition Examination Surveys 2010–2012 (*N* = 6055) was included in the logistic regressions.

**Results:**

The proportions of the respondents under- and over-perceiving their body weight were 66.9% and 0.8% among men and 16.3% and 15.6% among women respectively. A moderating effect of PA participation was observed on the relationship between body weight over-perception and depressed mood (Odds Ratio [OR] = 0.55; 95% Confidence Intervals [95% CI] = 0.34, 0.89). Among individuals who did not meet the recommended vigorous-intensity PA (≥ 20 min/session and ≥ 3 day/week), body weight over-perception was associated with depressed mood (OR = 1.71, 95% CI = 1.19, 2.46) compared to the accurate-perception group. However, no association was observed among those who met the recommended vigorous-intensity PA (OR = 1.52, 95% CI = 0.45, 5.22). Similar patterns were found among physically active versus inactive individuals (recommended walking not met: OR = 2.02, 95% CI = 1.29, 3.15; recommended walking met: OR = 1.28, 95% CI = 0.66, 2.49; muscular strengthening exercises for < 2 day/week: OR = 1.74, 95% CI = 1.21, 2.51; muscular strengthening exercises for ≥ 2 day/week: OR = 1.38, 95% CI = 0.37, 5.14). No relationship existed between body weight over-perception and depressed mood after adjusting for PA.

**Conclusions:**

Participating in regular PA may buffer a potential negative impact of body weight over-perception on depressive mood.

## Background

The physical co-morbidities associated with obesity are well known (e.g., type 2 diabetes mellitus, cardiovascular disease, dyslipidemia, hypertension) [1, 2], yet the relationship between obesity and psychological health is still not fully understood. Some previous studies found that obesity is related to increased psychological distress with inconsistent results by gender [3] and/or the degree of obesity [4, 5], while others have found no [6] or protective association [7]. It may be that body image or perception of body weight, rather than actual body weight, is more strongly associated with psychological distress. This is, in part, evidenced by accumulating research with different population groups. For example, body weight misperception (i.e., discrepancy between individual perceptions of weight status and actual weight status based on clinical definitions of weight) [8] has been reported among individuals who are and are not affected by obesity and may be associated with various forms of psychological distress including stressed feelings, depressive mood, and suicidal ideation [8–10]. Recent studies with South Korean adults suggest that body weight misperception, over-perception (i.e., over-perceiving actual weight status) in particular, is associated with psychological distress including severe stress and depressed mood in women [11–13], and suicidal ideation in both genders [14, 15].

Body weight perception reflects sociocultural aspects of desired body size, which change over time [16, 17]. In South Korea, being plump was traditionally considered the ideal body type, since it exemplified good health, wealth, and high social class [17]. However, rapid westernization of the country since the 1970s has led to changes in sociocultural norms of the ideal body type from plump to slender. This shift in ideal body type influences young South Korean adults to under-estimate or over-estimate their body weight and to subsequently engage in unhealthy behaviours in attempting to control their weight due to social desirability [17–20]. For example, in a study examining body image and weight control practices among university students from 22 countries, 43% of South Korean female participants perceived themselves as overweight and 77% were attempting to lose weight, while the mean body mass index (BMI) score (19.3 kg/m^2^) was the lowest in the study [20]. Also, though body weight over-perception (14%) and weight control attempts (23%) were less severe in young South Korean males compared to their female counterparts, the proportion of South Korean males who try to lose weight was high relative to their male counterparts in different countries (8^th^), while their mean BMI score (20.7 kg/m^2^) was the second lowest among the 22 countries [20].

In South Korea, the prevalence of weight misperception appears to be elevated in young adults relative to older adults [11, 17]. Though the existing research has primarily focused on women, body weight under-perception is prevalent in young male adults aged 20 to 39 years and they were most likely to attempt to maintain or gain weight compared to all other age groups [17]. Furthermore, among adolescents, males who underestimate and females who overestimate their body weight were more likely to report depressed feelings than those with accurate body weight perception [21]. These results suggest that body weight misperception is a weight-related issue that is not mutually exclusive to women. In addition, though patterns may vary by gender, body weight misperception is associated with psychological distress [15, 17, 21]. Therefore, it is important to include both genders when exploring the relationship between body weight misperception and psychological distress.

In contrast to body weight misperception, which appears to have negative effects on psychological health, physical activity (PA) is positively associated with improved mood and health-related quality of life, as well as decreased levels of stress, depression, and anxiety [21, 22]. To achieve health benefits, the World Health Organization’s global PA guidelines recommend that adults should engage in a minimum of 150 min per week of moderate-intensity PA [23]. Given the well-established benefits of PA on psychological health, it is possible that individuals meeting PA guidelines are protected from developing psychological distress even if they misperceive their weight. However, a potential role of meeting PA guidelines on the association between body weight misperception and psychological distress has not yet been determined.

Therefore, the primary purpose of this study was to determine whether engaging in sufficient levels of PA (i.e., meeting vs. not meeting the PA guidelines) modifies the association between body weight misperception and psychological distress in a nationally representative sample of young South Korean adults. If PA was found to moderate the relationship between misperception and psychological distress, the secondary purposes were to investigate: 1) the associations between body weight misperception and psychological distress stratified by PA (i.e., meeting or not meeting the PA guidelines), and 2) the associations between body weight misperception and psychological distress after adjusting for PA to determine if the latter served as a confounder.

## Methods

### Study design and population

This study was conducted using data obtained from the 2010–2012 Korean National Health and Nutrition Examination Survey (KNHANES-V), an annual survey conducted by the Korea Centre for Disease Control and Prevention (KCDC). Young South Korean adults (*n* = 7740) aged 20 to 39 were included in the analysis. The KNHANES is a cross-sectional, nationally representative survey of the Korean population using a stratified, multistage probability sampling design for the selection of residence based on age, gender, and 13 metropolitan and non-metropolitan geographic locations. It includes a physical health examination and three self-report questionnaires (i.e., Health Behavior, Health Interview, and Nutrition) [24]. Detailed information about the survey is described elsewhere [24]. Among those who participated in the survey, 6055 young adults (45.1% female) were included in the analysis after excluding individuals who felt physical pain or discomfort due to chronic/acute diseases or accidents over the previous two weeks (*n* = 976) and those with missing data (*n* = 709; 9.2%). No significant differences existed in the distribution of age and gender before and after exclusions (*p* > 0.05). Identifying information was removed when the KCDC released the data [25].

### Measures

#### Body weight misperception

Body weight misperception was defined as a mismatch between an individual’s measured weight status based on BMI and self-reported weight status. Based on the objectively measured weight (kilograms [kg]) and height (meters [m]), BMI (kg/m^2^) scores for all respondents were calculated. The Asia Pacific Islanders BMI cut-offs recommended by the World Health Organization, International Obesity Task Force, and Korean Society of the Study of Obesity were used to determine actual body weight status [26, 27]. In this study, BMI cut-offs are: underweight (BMI < 18.50); normal weight (BMI 18.50–22.99); overweight (BMI 23.00–24.99); and obese (BMI ≥ 25). Perceived body weight was assessed using the multiple choice question, ‘What do you think about your body?’ with the possible answers, ‘very underweight’, ‘slightly underweight’, ‘normal’, ‘slightly overweight’, and ‘very overweight’. Perceived body weight status was re-categorized into four groups: very thin/slightly thin (combined due to low frequencies), normal, slightly fat, and very fat. Body weight under-perception occurred when participants reported their body weight being: 1) thin but their BMI placed them in the normal or overweight category, and 2) normal weight but their BMI placed them in the overweight category. Body weight over-perception occurred when participants reported their body weight being: 1) normal or overweight but their BMI placed them in the underweight category, or 2) slightly or very fat but their BMI placed them in the normal weight category. Respondents with consistency between actual and perceived body size were categorized as the accurate body weight perception group.

#### Psychological distress

Three dimensions of psychological distress were assessed with questions about stress, depressed mood, and suicidal ideation. To measure stress, participants were asked to rate their level of daily stress on a four-point scale, which was then dichotomized to reflect ‘low stress’ for the responses ‘rarely feel stressed’ and ‘a little bit stressed’, and ‘high stress’ for the responses ‘feel quite stressed’ or ‘feel extremely stressed.’ Depressed mood was measured by asking participants whether they felt depressed for two consecutive weeks. Finally, suicidal ideation was measured by asking participants whether they had thought about committing suicide during the past year. The possible responses for depressed mood and suicidal ideation were ‘yes’ or ‘no’. These measures were reported as predictors of psychological distress in previous studies [11, 13, 14, 28].

#### Physical activity (PA)

The International Physical Activity Questionnaire (IPAQ) was used to measure PA [29]. Participants were asked how many days over the course of the previous seven days had they engaged in: vigorous-intensity PA (VPA) for more than 10 min; moderate-intensity PA (MPA), which excluded walking, for more than 10 min; walking for more than 10 min; and muscle strengthening exercises (MSE).

Participants were also asked to indicate how long per day (hours and minutes) they had engaged in each type of PA. Responses were scaled from 1 (no participation at all) to 8 (7 day/week) for VPA, MPA, and walking. In this study, PA and physical inactivity were determined based on the reported VPA, MPA, and walking generated by the KNHANES. Specifically, meeting the recommended levels of VPA, MPA, and walking were defined as: a) participated in VPA ≥ 30 min/session for ≥ 3 day/week; b) participated in MPA ≥ 30 min/session for ≥ 5 day/week; and c) participated in walking ≥ 30 min/session for ≥ 5 day/week, respectively. Those who did not meet the recommended levels of VPA, MPA, and walking were coded as ‘0.’ MSE was initially scored from 1 (no participation) to 4 (≥ 5 day/week), but was re-categorized into ‘0 (< 2 day/week)’ and ‘1 (≥ 2 day/week)’ because some categories had low frequency. Furthermore, one overall PA variable was generated based on the VPA, MPA, walking, and MSE variables. Specifically, the binary overall PA variable was coded as ‘1 (meeting any type of PA recommendations)’ and ‘0 (not meeting any type of PA recommendations).

#### Covariates

Socioeconomic, demographic, and lifestyle variables were included as covariates. Socioeconomic variables were equivalized household income, education, and employment status. Monthly equivalized household income was categorized by quartile. Educational attainment was categorized into elementary or less (≤ 6 years), middle school (7–9 years), high school (10–12 years), and college and above (≥ 13 years), then re-categorized into two groups for the analyses: high school graduate or less and post-secondary graduate or more. Employment status was categorized as employed and unemployed, where unemployed included students and housewives. Demographic variables included age, gender, marital status and area of residence. Marital status was categorized as never married, married and living with a spouse, and previously married (i.e., divorced, separation by death, or other). Area of residence was divided into Metro Seoul (Seoul, Incheon metropolitan cities, and Gyeonggi province) and Non-Metro Seoul, since social and health inequalities between these two regions have been reported [30]. Lifestyle covariates included Self-Rated Health (SRH), smoking, and alcohol consumption. SRH was categorized as good, fair, and bad. Smoking was categorized as currently smoking or not. With respect to alcohol consumption, participants were classified into high and low risk groups based on guidelines provided by KNHANES (high risk was defined as ≥ seven cups at a single event and ≥ twice a week for males, and ≥ five cups at a single event and ≥ twice a week for females) [25].

### Statistical analyses

Population weights provided by KNHANES were applied to all analyses to account for the complex survey design. Descriptive statistics were calculated to estimate means and standard deviations, or percentages. Main and moderating effects of body weight misperception and PA on psychological distress (i.e., stressed feelings, depressive mood, and suicidal ideation) were examined by entering separate interaction terms to logistic regression models [31]. Significant interaction terms would indicate a moderating effect of PA on the relationship between body weight misperception and psychological distress and justify stratifying the analyses by PA. Logistic regressions were then performed for individuals who met the recommended level of VPA, MPA, and walking versus individuals who did not; and who participated in MSE for ≥ 2 day/week vs. < 2 day/week. No adjustments were made for these analyses. Finally, multiple logistic regression models were also conducted to examine the associations between body weight misperception and psychological distress before (unadjusted) and after adjusting for covariates (Models 1–3) and PA (Model 4). Model 1 accounted for age and gender; Model 2 adjusted for age, gender, household income, education level, employment status, marital status, and area of residence; Model 3 additionally adjusted for SRH, smoking, and alcohol consumption. Finally, Model 4 adjusted for all covariates and PA levels. For the final regression model, PA was dichotomized as meeting any of PA guidelines versus not meeting any. Of note, a series of logistic regression analyses were conducted with the inclusion of two-way interaction terms in the models to test whether the relationships between body weight misperception and psychological distress vary between males and females. However, no significant gender-by-weight misperception interactions were observed; thus, gender-stratified analyses were not conducted. The results were reported as odds ratios (OR) and 95% confidence intervals (95% CI). All analyses were completed using SAS version 9.4 with an alpha level of 0.05.

## Results

Demographic characteristics of the study sample and by gender are presented in Table 1. Over one quarter (27.1%) of participants were categorized as obese (35.9% of males and 17.1% of females). A total of 47.0% of men and 37.7% of women reported being healthy. Over half the sample misperceived their body weight (51.1%), with more inaccurate perceptions among men (67.8%) than women (32.0%). The proportion of the sample under-perceiving their body weight was higher among men (66.9%) than women (16.3%), whereas the proportion over-perceiving their body weight was higher among women (15.6%) compared to men (0.8%). With respect to psychological distress, 27.8% of men and 34.4% of women reported feeling stressed, 6.8% of men and 12.7% of women reported feeling depressed, and 6.5% of men and 16% of women reported considering suicide in the past year.

**Table 1:** Descriptive characteristics of a sample of young South Korean adults aged 20 to 39 years --2010–12 Korea National Health and Nutrition Examination Survey (KNHANES) (*N* = 6055)

	Total (Mean ± SD) (*N* = 6055)	Men (Mean ± SD) (*n* = 2572)	Women (Mean ± SD) (*n* = 3483)
Age (years)	30.06 ± 5.5	29.93 ± 5.5	30.21 ± 5.5
Body mass index (kg/m^2^)	23.17 ± 3.7	24.20 ± 3.6	21.99 ± 3.5
	Total (%)	Men (%)	Women (%)
Education			
≥ Post-sec graduate	47.9	50.3	45.1
Household income^a^			
Quartile 1	8.3	8.9	7.6
Quartile 2	27.7	27.1	28.3
Quartile 3	35.0	35.5	34.3
Quartile 4	29.1	28.4	29.7
Marital status			
Never married	49.9	56.5	42.2
Married living with spouse	48.3	42.5	55.0
Previously married^b^	1.8	1.0	2.8
Employment status (unemployed)^c^	34.1	22.2	47.5
Area of residence^d^			
Metro Seoul	29.5	28.5	30.7
Non-Metro Seoul	70.5	71.5	69.3
Self-rated health			
Healthy	42.7	47.0	37.7
Neutral	48.4	45.1	52.1
Not healthy	8.9	7.9	10.2
Currently smoking	32.3	52.9	8.9
High-risk drinking^e^	16.9	23.0	10.0
Physical activity			
Vigorous-intensity ≥ 20 min/session, ≥ 3 day/week	15.5	19.5	11.0
Moderate-intensity ≥ 30 min/session, ≥ 5 day/week	9.5	11.7	6.9
Walking ≥ 30 min/session, ≥ 5 day/week	43.5	47.0	39.4
Muscular strengthening ≥ 2 day/week	22.5	32.3	11.2
Body Mass Index (kg/m^2^)			
Underweight < 18.5	7.5	2.9	12.7
Normal 18.5–22.9	39.3	30.5	49.2
Overweight 23–24.9	26.2	30.6	21.0
Obese ≥ 25	27.1	35.9	17.1
Body weight perception			
Under-perception	43.3	66.9	16.3
Accurate-perception	48.9	32.2	68.0
Over-perception	7.7	0.8	15.6
Psychological distress			
Stressed	30.9	27.8	34.4
Depressed	9.5	6.8	12.7
Suicidal ideation	10.9	6.5	16.0

^a^Income quartiles were calculated based on standardized income (total monthly household income divided by the square root of the number of household members)

^b^Previously married included individuals who are divorced or separated from their spouse by death or other reasons

^c^Unemployed status included students and housewives

^d^Metro Seoul included Seoul, Incheon metropolitan cities, and Gyeonggi province

^e^High-risk drinking: ≥ 7 cups/occasion and ≥ twice/week for men; ≥ 5 cups/occasion and ≥ twice/week for women [23]

The main and moderation effects of body weight misperception and PA on three psychological distress measures are shown in Table 2. Body weight under-perception was associated with a lower likelihood of reporting depressed mood (OR = 0.50; 95% CI = 0.34, 0.74) and suicidal ideation (OR = 0.48, 95% CI = 0.36, 0.63). However, no moderation effect existed for the association between body weight under-perception and any of the three markers of psychological distress. Body weight over-perception was associated with a higher likelihood of depressed mood (OR = 2.69, 95% CI = 1.96, 3.69). PA participation (i.e., meeting PA recommendations) was found to modify the relationship between body weight over-perception and depressed mood (OR = 0.55; 95% CI = 0.34, 0.89). No other main or interaction effects existed.

**Table 2:** Unadjusted main and interaction effects of body weight misperception and physical activity on psychological distress among young South Korean adults aged 20 to 39 years—2010–12 Korea National Health and Nutrition Examination Survey (KNHANES) (*N* = 6055)

	Stressed feeling	Depressed mood	Suicidal ideation
	OR (95% CI)	OR (95% CI)	OR (95% CI)
Body weight under-perception	0.87 (0.72, 1.01)	0.50 (0.34, 0.74)*	0.48 (0.36, 0.63)*
Physical activity^a^	0.98 (0.85, 1.12)	0.83 (0.62, 1.11)	0.88 (0.72, 1.06)
Body weight under-perception* physical activity^a^	0.95 (0.76, 1.19)	1.00 (0.58, 1.73)	1.41 (0.90, 1.90)
Body weight over-perception	1.50 (1.17, 1.93)*	2.69 (1.96, 3.69)*	2.06 (1.49, 2.85)*
Physical activity^a^	0.96 (0.85, 1.08)	0.90 (0.74, 1.08)	0.92 (0.77, 1.10)
Body weight over-perception* physical activity^a^	0.94 (0.66, 1.33)	0.55 (0.34, 0.89)*	0.95 (0.60, 1.50)

^a^Meeting any type of recommended level of physical activity

*p < 0.05

The associations between body weight misperception and depressed mood by PA strata (active vs. inactive) are shown in Table 3 (reference group: accurate body weight perception). Overall, body weight under-perception was associated with lower likelihoods of depressed mood regardless of PA levels except for those who did not meet the MPA recommendation. Specifically, body weight under-perception was associated with lower likelihoods of depressed mood among individuals who are physically active (VPA: OR = 0.43, 95% CI = 0.22, 0.85; walking: OR = 0.51, 95% CI = 0.32, 0.84; MSE: OR = 0.39, 95% CI = 0.20, 0.77) and inactive (VPA: OR = 0.55, 95% CI = 0.40, 0.77; MPA: OR = 0.55, 95% CI = 0.41, 0.76; walking: OR = 0.55, 95% CI = 0.38, 0.79; MSE: OR = 0.59, 95% CI = 0.42, 0.83). No association existed between body weight under-perception and depressed mood among individuals who met the MPA recommendation (OR = 0.33, 95% CI = 0.11, 1.03).

**Table 3:** Unadjusted associations between body weight misperception (reference group: accurate-perception) and depressed mood by physical activity levels among young South Korean adults aged 20 to 39 years—2010–12 Korea National Health and Nutrition Examination Survey (KNHANES) (*N* = 6055)

	Depressed mood	
	Under-perception	Over-perception
	OR (95% CI)	OR (95% CI)
Physical activity		
Recommended VPA not met^a^	0.55 (0.40, 0.77)*	1.71 (1.19, 2.46)*
Recommended VPA met	0.43 (0.22, 0.85)*	1.52 (0.45, 5.22)
Recommended MPA not met^b^	0.55 (0.41, 0.76)*	1.55 (1.06, 2.26)*
Recommended MPA met	0.33 (0.11, 1.03)	3.64 (1.09, 12.14)*
Recommended walking not met^c^	0.55 (0.38, 0.79)*	2.02 (1.29, 3.15)*
Recommended walking met	0.51 (0.32, 0.84)*	1.28 (0.66, 2.49)
MSE < 2 day/week^d^	0.59 (0.42, 0.83)*	1.74 (1.21, 2.51)*
MSE ≥ 2 day/week	0.39 (0.20, 0.77)*	1.38 (0.37, 5.14)

Referent group: Accurate weight perception

^a^Recommended VPA (vigorous-intensity physical activity) ≥ 20 min/session, ≥ 3 day/week

^b^Recommended MPA (moderate-intensity physical activity) ≥ 30 min/session, ≥ 5 day/week

^c^Recommended walking ≥ 30 min/session, ≥ 5 day/week

^d^ *MSE*: Muscular strengthening exercises

*p < 0.05

Among physically active individuals, no association existed between body weight over-perception and depressed mood except for those who met the recommended level of MPA. Specifically, among individuals who did not meet the recommended level of VPA (≥ 20 min/session and ≥ 3 day/week), those who over-perceive their body weight were more likely to report depressed mood (OR = 1.71, 95% CI = 1.19, 2.46). However, among those who participated in the recommended level of VPA, no association was observed between body weight over-perception and depressed mood (OR = 1.52, 95% CI = 0.45, 5.22). Similarly, among those who did not meet the recommended level of walking, body weight over-perception was associated with a higher likelihood of depressed mood (OR = 2.02, 95% CI = 1.29, 3.15) compared to accurate perception. No association existed for individuals who met the recommended level of walking (OR = 1.28, 95% CI = 0.66, 2.49). Lastly, among individuals who participated in MSE for < 2 day/week, body weight over-perception was associated with reporting a higher likelihood of depressed mood (OR = 1.74, 95% CI = 1.21, 2.51) compared to accurate perception. However, among those who participated in MSE for ≥ 2 day/week, no association was observed between body weight over-perception and depressed mood (OR = 1.38, 95% CI = 0.37, 5.14).

The associations of body weight over-perception with depressed mood before and after adjusting for covariates are shown in Table 4 (referent group: accurate body weight perception). Before entering covariates (unadjusted), individuals over-perceiving their body weight showed increased odds of depressed mood (OR = 1.68, 95% CI = 1.19, 2.39) compared to their accurate perception counterparts. After adjusting for age and gender (Model 1), individuals over-perceiving their body weight were still more likely to report feeling depressed (OR = 1.48, 95% CI = 1.04, 2.13) than those who accurately perceived their body weight. After adjusting for age, gender, household income, education levels, employment status, marital status, and area of residence (Model 2), body weight over-perception was associated with higher odds for depressed feeling (OR = 1.50, 95% CI = 1.04, 2.17). The association between body weight over-perception and depressed mood remained significant even after further adjusting for SRH, smoking, and alcohol consumption (Model 3; OR = 1.45, 95% CI = 1.01, 2.10). However, the association between body weight over-perception and depressed mood became insignificant when PA was entered in the final model (Model 4), which indicates the confounding effect of PA on this relationship.

**Table 4:** Unadjusted and adjusted associations between body weight misperception and the presence of depressed mood among young South Korean adults aged 20 to 39 years—2010–12 Korea National Health and Nutrition Examination Survey (KNHANES) (*N* = 6055)

	Depressed mood
	OR (95% CI)
Unadjusted	
Accurate-perception	1.00 (reference)
Under-perception	0.53 (0.39, 0.72)*
Over-perception	1.68 (1.19, 2.39)*
Model 1	
Accurate-perception	1.00 (reference)
Under-perception	0.66 (0.46, 0.95)*
Over-perception	1.48 (1.04, 2.13)*
Model 2	
Accurate-perception	1.00 (reference)
Under-perception	0.63 (0.44, 0.91)*
Over-perception	1.50 (1.04, 2.17)*
Model 3	
Accurate-perception	1.00 (reference)
Under-perception	0.70 (0.48, 1.02)
Over-perception	1.45 (1.00, 2.10)*
Model 4	
Accurate-perception	1.00 (reference)
Under-perception	0.72 (0.48, 1.02)
Over-perception	1.45 (0.99, 2.11)

Model 1: Adjusted for age and gender

Model 2: Adjusted for covariates in Model 1, household income, education level, employment status, marital status, and area of residence

Model 3: Adjusted for covariates in Model 2, self-rated health, smoking, and high risk drinking

Model 4: Adjusted for covariates in Model 3 and meeting any type of recommended level of physical activity

*p < 0.05

## Discussion

This study demonstrated that there are associations between body weight over-perception and three dimensions of psychological distress, namely depressive mood, stress, and suicidal ideation, among young South Korean adults. In addition to showing a main effect of body weight over-perception on psychological distress, we also identified a modifying role of PA on the association between body weight over-perception and depressive mood. That is, the association remained significant for inactive individuals, but became insignificant for those who engaged in sufficient levels of PA; indicating that engaging in PA may buffer against depressive mood among those who over-perceive their body weight. Participating in sufficient levels of PA may negate the potential negative influence of body weight over-perception on depressive mood. This makes sense given the well-established positive relationship between PA and psychological health [21, 22]. Future research examining the associations between body weight misperception and psychological health among the young adult population in South Korea should consider PA as an important effect modifier.

Our study also found that PA is a confounder on the association between body weight over-perception and depressed mood among young South Korean adults. This indicates that PA is associated with body-weight over perception, and an independent risk factor for depressed mood. This is another key finding of this study, since most other studies on this population have not taken into account PA. In one previous study examining the association between depressed mood and weight over-perception among South Korean women aged 20 to 34 years, the association became null after adjusting for PA [11]. Combined, PA should be included as a covariate when examining the relationship between body weight misperception and psychological distress in future research.

This study showed that PA is an effect modifier and a confounder on the association between body weight over-perception and depressed mood. While confounding is associated with both exposure and outcome, effect modification indicates the changes in magnitude of the effect of an exposure on an outcome by different levels of a third variable [32]. Though effect modification is distinct from confounding, dealing with the confounding or modifying effect of a third variable include either stratifying or adjusting for the third variable. As such, future research examining the relationship between body weight misperception and psychological distress among young South Korean adults should take PA into consideration to avoid spurious findings. These include stratifying analysis by or at least adjusting for different PA levels.

The findings related to suicidal ideation and stress are also consistent with previous studies that have suggested a potential association between weight over-perception and suicidal ideation in South Korean adults [14, 15]. In addition, a study conducted among South Korean adolescents found that a significant association between BMI and suicidal ideation was eliminated by including body weight perception, indicating a mediating effect, but no interaction effect of body weight over-perception and PA on suicidal ideation existed [33]. Similarly, an association existed between body weight over-perception and stress but no interaction effect of body weight over-perception and PA on stress was observed [15]. Though many factors are known to influence suicidal ideation and stress (e.g., demographics, health status, and health behaviours), body weight over-perception may be a prominent antecedent of such psychological distress regardless of PA levels. In another study, the association between high stress and body weight misperception sustained even after controlling for PA among South Korean women aged 20 to 64 years [11]. But, the association disappeared when other factors (i.e., SRH, depressed mood, suicide ideation) were further adjusted [11]. Clearly, more research with rigorous study design (e.g., longitudinal, experimental) is required to determine the association between body weight misperception and psychological distress.

Unlike body weight over-perception, body weight under-perception was associated with lower odds of stressed feeling, depressed mood, and suicidal ideation among young South Korean adults. In addition, body weight under-perception was consistently associated with lower odds of depressed mood even when analyses were stratified by PA levels (i.e., active vs. inactive). The contemporary ideal body type (i.e., being slender) and corresponding social desirability may play a part in reducing or eliminating weight-related psychological issues among those who under-perceive their body weight [17–20]. Furthermore, given that the adverse association between body weight under-perception and depressed mood became null after adjusting for SRH, smoking, and high risk drinking, these variables may be more relevant to psychological distress, than PA, among individuals with body weight under-perception.

A main strength of this study is its large, nationally representative sample of young South Korean adults, along with a complex sampling technique to obtain an accurate depiction of the South Korean population. Also, we were able to directly measure height and weight, which provided reliable BMI. Additionally, the inclusion of males in the analysis allowed a more comprehensive assessment. Several studies conducted in South Korea have only examined the relationship between body weight misperception and psychological distress among women because they are considered an “at-risk population” on weight-related psychological issues [11, 18]. Our findings show that the relationship between body weight over-perception and depressive mood remained significant after adjusting for gender, indicating that males should not be excluded from future studies. Finally, this study considered an individual’s perception of his/her body weight rather than actual weight, which may have a larger role in psychological health.

Still, there are some limitations to report. The cross-sectional design does not allow for identifying whether body weight perception occurred before psychological distress, therefore causal inference is not possible. However, our research question has intuitive appeal. This limitation has been identified in most studies investigating the relationship between body weight perception and psychological distress. Future research should employ longitudinal designs to better understand this relationship and the factors affecting it. Furthermore, there are drawbacks to relying on self-reported PA and psychological distress. Although the IPAQ is a reliable tool [29], individuals may still over-report their PA levels [34]. Also, psychological distress was determined from only a few simple questions rather than an in-depth psychological analysis. These self-reported answers may have been subject to social desirability bias, especially given the widespread stigmatization of mental illness in Asian countries [35]. That being said, it would not have been feasible to administer comprehensive psychological tests via self-report, but we acknowledge that dichotomizing the measures of psychological distress likely oversimplified a complex phenomenon. Lastly, we used the single-item measured body weight perception variable and BMI to generate body weight misperception. In previous literature, body weight misperception has been used interchangeably with body size misperception, body image distortion, misclassification of one’s weight and/or subjective body image misperception (e.g., [10–15, 36, 37]). It will be informative for future research to theoretically distinguish these terms and define how they each can be conceptualized and operationalized.

## Conclusions

Body weight over-perception was associated with an increased risk for reporting being in a depressed mood in physically inactive young South Korean adults, but not for those who met PA recommendations. This provides further evidence for the positive influence of PA on psychological health and well-being, and supports the need for increased promotion of PA in South Korea. It remains to be determined if increasing PA alleviates the burden of body weight misperception on psychological distress. Future studies should also consider why individuals misperceive their weight and develop strategies to improve young adults’ weight perception.

## Abbreviations

BMI: Body mass index
CI: Confidence interval
IPAQ: International Physical Activity Questionnaire
KCDC: Korea Center for Disease Prevention and Control
KNHANES: Korea National Health and Nutrition Examination Survey
MPA: Moderate-intensity physical activity
MSE: Muscle strengthening exercises
OR: Odd ratio
SRH: Self rated health
VPA: Vigorous-intensity physical activity

## References

[1] Dixon JB (2010). The effect of obesity on health outcomes. Mol Cell Endocrinol.

[2] Kopelman P (2007). Health risks associated with overweight and obesity. Obes Rev.

[3] Carpenter KM, Hasin DS, Allison DB, Faith MS (2000). Relationships between obesity and DSM-IV major depressive disorder, suicide ideation, and suicide attempts: results from a general population study. Am J Public Health.

[4] Roberts RE, Kaplan GA, Shema SJ, Strawbridge WJ (2000). Are the obese at greater risk for depression?. Am J Epidemiol.

[5] Luppino FS, de Wit LM, Bouvy PF, Stijnen T, Cuijpers P, Penninx BW, Zitman FG (2010). Overweight, obesity, and depression: a systematic review and meta-analysis of longitudinal studies. Arch Gen Psychiatry.

[6] Robert ER, William JS, Stephane D, George AK (2002). Are the fat more jolly?. Ann Behav Med.

[7] Jorm AF, Korten AE, Christensen H, Jacomb PA, Rodgers B, Parslow RA (2003). Association of obesity with anxiety, depression and emotional well‐being: a community survey. Aust N Z J Public Health.

[8] Atlantis E, Ball K (2008). Association between weight perception and psychological distress. Int J Obes.

[9] Ter Bogt TF, van Dorsselaer SA, Monshouwer K, Verdurmen JE, Engels RC, Vollebergh WA (2006). Body mass index and body weight perception as risk factors for internalizing and externalizing problem behavior among adolescents. J Adolesc Health.

[10] Krauss RC, Powell LM, Wada R. Weight misperceptions and racial and ethnic disparities in adolescent female body mass index. J Obes. 2012. doi:10.1155/2012/205393. 10.1155/2012/205393 PMC3371348 22701166

[11] Choi Y, Choi E, Shin D, Park SM, Lee K (2015). The association between body weight misperception and psychosocial factors in Korean adult women less than 65 years old with normal weight. J Korean Med Sci.

[12] Kim DS, Kim HS, Cho Y, Cho SI (2008). The effects of actual and perceived body weight on unhealthy weight control behaviors and depressed mood among adult women in Seoul, Korea. J Prev Med Public Health.

[13] Lee KM, Seo MS, Shim JY, Lee YJ (2015). Body weight status misperception and its association with weight control behaviours, depressive mood and psychological distress in nulliparous normal-weight young women. Ann Hum Biol.

[14] Shin J, Choi Y, Han KT, Cheon SY, Kim JH, Lee SG, Park EC (2015). The combined effect of subjective body image and body mass index (distorted body weight perception) on suicidal ideation. J Prev Med Public Health.

[15] Chin YR, Lee HY, So ES (2011). Suicidal ideation and associated factors by sex in Korean adults: a population-based cross-sectional survey. Int J Public Health.

[16] Burke MA, Heiland FW, Nadler CM (2010). From “overweight” to “about right”: evidence of a generational shift in body weight norms. Obesity.

[17] Joh HK, Oh J, Lee HJ, Kawachi I (2013). Gender and socioeconomic status in relation to weight perception and weight control behavior in Korean adults. Obes Facts.

[18] Kim M, Lee H (2010). Overestimation of own body weights in female university students: associations with lifestyles, weight control behaviors and depression. Nutr Res Pract.

[19] Lim H, Wang Y (2013). Body weight misperception patterns and their association with health‐related factors among adolescents in South Korea. Obesity.

[20] Wardle J, Haase AM, Steptoe A (2006). Body image and weight control in young adults: international comparisons in university students from 22 countries. Int J Obes.

[21] Penedo FJ, Dahn JR (2005). Exercise and well-being: a review of mental and physical health benefits associated with physical activity. Curr Opin Psychiatry.

[22] Anokye NK, Trueman P, Green C, Pavey TG, Taylor RS. Physical activity and health related quality of life. BMC Public Health. 2012;12(624). 10.1186/1471-2458-12-624 PMC3490805 22871153

[23] World Health Organization (2010). Global recommendations on physical activity for health.

[24] Kweon S, Kim Y, Jang MJ, Kim Y, Kim K, Choi S, Chun C, Khang YH, Oh K (2014). Data resource profile: the Korea national health and nutrition examination survey (KNHANES). Int J Epidemiol.

[25] Korea Centers for Disease Control and Prevention (2012). The Fifth Korea National Health and Nutrition Examination Survey (KNHANES V-3).

[26] Oh SW (2011). Obesity and metabolic syndrome in Korea. Diabetes Metab J.

[27] World Health Organization (2004). International statistical classification of diseases and related health problems.

[28] Kim IH, Muntaner C, Khang YH, Paek D, Cho SI (2006). The relationship between nonstandard working and mental health in a representative sample of the South Korean population. Soc Sci Med.

[29] Craig CL, Marshall AL, Sjöström M, Bauman AE, Booth ML, Ainsworth BE, Pratt M, Ekelund U, Yngve A, Sallis JF, Oja P (2003). International Physical Activity Questionnaire (IPAQ): 12-country reliability and validity. Med Sci Sports Exerc.

[30] Hwang J, Shon C (2014). Relationship between socioeconomic status and type 2 diabetes: results from Korea National Health and Nutrition Examination Survey (KNHANES) 2010–2012. BMJ Open.

[31] Hayes AF, Matthes J (2009). Computational procedures for probing interactions in OLS and logistic regression: SPSS and SAS implementations. Behav Res Methods.

[32] Kim DS, Cho Y, Cho SI, Lim IS (2009). Body weight perception, unhealthy weight control behaviors, and suicidal ideation among Korean adolescents. J Sch Health.

[33] Bauman AE, Sallis JF, Dzewaltowski DA, Owen N (2002). Toward a better understanding of the influences on physical activity: the role of determinants, correlates, causal variables, mediators, moderators, and confounders. Am J Prev Med.

[34] Adams SA, Matthews CE, Ebbeling CB, Moore CG, Cunningham JE, Fulton J, Hebert JR (2005). The effect of social desirability and social approval on self-reports of physical activity. Am J Epidemiol.

[35] Lauber C, Rössler W (2007). Stigma towards people with mental illness in developing countries in Asia. Int Rev Psychiatry.

[36] Lee J, Lee Y. The association of body image distortion with weight control behaviors, diet behaviors, physical activity, sadness, and suicidal ideation among Korean high school students: a cross-sectional study. BMC Public Health. 2016;16(39). 10.1186/s12889-016-2703-z PMC4714421 26772963

[37] Quick V, Nansel TR, Liu D, Lipsky LM, Due P, Iannotti RJ (2014). Body size perception and weight control in youth: 9-year international trends from 24 countries. Int J Obes.

